# Characterization of Bulgarian Copper Mine Tailing as a Precursor for Obtaining Geopolymers

**DOI:** 10.3390/ma17030542

**Published:** 2024-01-23

**Authors:** Darya Ilieva, Lyudmila Angelova, Temenuzhka Radoykova, Andriana Surleva, Georgi Chernev, Petrica Vizureanu, Dumitru Doru Burduhos-Nergis, Andrei Victor Sandu

**Affiliations:** 1 Faculty of Chemical Technologies, University of Chemical Technology and Metallurgy, 8 “St. Kl. Ohridski” Blvd., 1756 Sofia, Bulgaria; daryailieva@abv.bg (D.I.); lyudmila@uctm.edu (L.A.); nusha_v@uctm.edu (T.R.); 2 Faculty of Metallurgy and Materials Science, University of Chemical Technology and Metallurgy, 8 “St. Kl. Ohridski” Blvd., 1756 Sofia, Bulgaria; g.chernev@uctm.edu; 3 Faculty of Materials Science and Engineering, “Gheorghe Asachi” Technical University of Iasi, 67 Prof. D. Mangeron Blvd., 700050 Iasi, Romania; doru.burduhos@tuiasi.ro (D.D.B.-N.); sav@tuiasi.ro (A.V.S.); 4 Technical Sciences Academy of Romania, 26 Dacia Blvd., 030167 Bucharest, Romania

**Keywords:** mine tailing, geopolymers, alkaline reactivity, characterization, heavy metals, leaching tests, valorization, raw materials

## Abstract

Valorization of high-volume mine tailings could be achieved by the development of new geopolymers with a low CO_2_ footprint. Materials rich in aluminum and silicon with appropriate solubility in an alkaline medium can be used to obtain a geopolymer. This paper presents a study of copper mine tailings from Bulgaria as precursors for geopolymers. Particle size distribution, chemical and mineralogical composition, as well as alkaline reactivity, acidity and electroconductivity of aqueous slurry are studied. The heavy metal content and their mobility are studied by leaching tests. Sequential extraction was applied to determine the geochemical phase distribution of heavy metals. The studied samples were characterized by high alkalinity, which could favor the geopolymerization process. The water-soluble sulphates were less than 4%. The Si/Al ratio in mine tailing was found to be 3. The alkaline reactivity depended more so on the time of extraction than on the concentration of NaOH solution. The main part of the heavy metals was found in the residual fraction; hence, in high alkaline medium during the geopolymerization process, they will stay fixed. Thus, the obtained geopolymers could be expected to exert low environmental impact. The presented results revealed that studied copper mine tailing is a suitable precursor for geopolymerization.

## 1. Introduction

Transformation of industrial wastes into a resource is one of the pillars of the circular economy. The need of waste valorization is widely recognized, and a large amount of effort is focused on the research and development of industrial processing activities for reusing or recycling industrial wastes. The mine activities and metal extraction processes generate an enormous amount of tailings usually stored in tailing dumps [1,2]. The disadvantages of this practice are well recognized, and the industry is eager to find alternatives to landfill practice [2,3].

Geopolymer technology offers a valuable approach to reuse mine tailings as well as other types of industrial wastes by applying low CO_2_ footprint processes. An advantage of the geopolymerization process is that the new composite materials could be obtained by fine tuning the precursor mixture containing low and high reactive silica and alumina to obtain a material with desired characteristics [4,5]. The blending of raw material mixtures allowed in some cases for the skipping of the activation step of low reactive precursors, thus ensuring eco-friendly process [4]. Coal combustion byproducts, blast furnace slag, fly ash, glass fibers, metakaolin, etc. were studied as raw materials with high reactive Al and Si [4,5]. The thorough characterization of mine tailing is required in order to finely adjust the technology to the particular characteristics of raw materials. Due to the specific chemical composition and characteristics of mine tailings from different sources, a deep study is needed for each tailing source. The characterization strategy should include not only the required characteristics for the obtaining technology, but also the assessment of some parameters, taking into account the application of the final product as well as the generated flows into the environment during its production, usage and post-application fate.

Over 55 billion cubic meters of mine tailings (MTs) are stored globally, with a 23% increase by 2025 [3]. Many storage facilities are vulnerable, with over 100 large dam collapses since 1960 causing death and environmental consequences [6]. Utilizing mine tailings as a replacement can conserve natural resources for 4 to 5 years, reducing the use of virgin raw materials in concrete production. Zhang et al. [7] explored the use of mine tailings as precursors for geopolymers, partially replacing fly ash (FA) with molybdenum tailings for cost-effective waste valorization. However, a higher percentage of FA substitution with MT leads to mechanical performance loss due to an increased macropore volume fraction. Qing et al. [8] produced geopolymer concrete with a compressive strength of 47.6 MPa using the alkali-hydrothermal activation of quartz powder at 300 °C. Although it meets 42.5 cement standards, high temperatures may hinder its sustainability. Orozco et al. [9] developed sustainable bricks by activating gold mine tailings with NaOH or (CaOH)_2_ and curing at 80 °C. Opiso et al. [10] found that adding 10% palm oil fuel ash to gold mine tailings-based geopolymer bricks improved their mechanical qualities and allowed for room-temperature curing, resulting in cost and CO_2_ emission savings. However, creating geopolymers with suitable mechanical characteristics using MTs as raw materials is challenging due to the large number of nonreacting phases, particularly quartz. Vizureanu et al. [6] also concluded that the properties of the geopolymers that use mine tailings as pre-cursors strongly depend on the characteristics of the raw materials. Next to the characteristics of the mine tailings, the activator parameters (type and concentration) and the curing conditions (temperature and time) will also affect the performance of the geopolymers. Krishna et al. [5] found low reactivity of metallurgical tumbling in mine tailings for a geopolymer manufacture. They suggested a customized processing approach for each location and available MT, considering factors like raw material properties, activation parameters and end product type.

Geopolymers based on copper mine tailings with and without blending with additional sources of aluminosilicates have been proposed for potential use as construction materials (bricks, pavements, road construction, etc.) or as an approach for hazardous element encapsulation [11,12,13,14,15,16,17,18]. As copper tailings showed low reactivity [12,17], preliminary activation was proposed, such as mechanical activation [16] or curing at elevated temperatures [11,13,16]. Castillo et al. [12] reported that heating at 90 °C promoted the dissolution of aluminosilicates in an alkaline medium and favored geopolymer hardening processes based on copper tailing. In contrast, Tian et al. [13] found that moderate heating (80 °C) promoted the dissolution of aluminosilicates, whereas higher temperatures negatively influenced the microstructure of the obtained geopolymers. The optimal curing temperature should be established empirically for the given raw materials and should depend on their reactivity and alkali concentration [11]. Geopolymer materials appropriate for low-strength applications were developed based on copper tailing and alkali activated fly ash or low-calcium slag as additional sources of reactive aluminosilicates [14,16]. High-calcium additives were reported to result in better characteristics of the obtained geopolymers [17]. A geopolymer concrete based on copper tailing and blast furnace slag was reported to show promising behavior in marine-related environments [18].

Mine tailing dumps in Bulgaria are well studied from a environmental or technological point of view. Some results on mine tailing valorization have been recently discussed in detail [19,20,21]. However, the mine tailing potential for application as raw materials for geopolymer obtaining is scarcely reported. The main challenges for the incorporation of mine tailings into geopolymer manufacturing are related to heavy metal contaminations and leaching. Wang et al. [22] reviewed the literature on geopolymers containing heavy metals and found that the process produces safe and long-lasting products through physical encapsulation, covalent bonding, ion exchange, and compound creation mechanisms. However, experimental validation is necessary due to the particularities of each mine tailing and geopolymer manufacturing method, especially when blended systems are researched.

The aim of the present study is characterization of copper mine tailing as a raw material for obtaining geopolymers with a low CO_2_ footprint. To achieve this goal, a specific algorithm for the chemical characterization of the item was developed to test not only the chemical composition parameters, but also the behavior of potentially hazardous components and their mobility. From a geopolymer technology point of view, the following parameters were studied: particle size distribution, chemical and mineralogical composition, characteristics of aqueous leachate, and alkaline reactivity. From an environmental point of view, the following characteristics were studied: heavy metal fractionalization, the composition of water leachate, and the mobility of hazardous components. The results revealed that the copper mine tailing from the Assarel Concentrator plant in Bulgaria has a great potential for valorization by using it as a precursor for obtaining geopolymers. This study presents for the first time a detailed characterization of copper mine tailing from Assarel, Bulgaria as a raw material for geopolymers obtained by alkali activation and low CO_2_ footprint technology.

## 2. Materials and Methods

### 2.1. Raw Materials Analysis

Copper mine tailings have been collected from the Assarel Concentrator plant in Bulgaria and comprehensively evaluated to establish its suitability as a precursor for geopolymers. The fly ash used in the study was from the burning of sub-bituminous coal in thermal power plants (TPP ”Bobov dol”, Bulgaria) and could be classified as class C (Table 1).

#### 2.1.1. Particle Size Distribution, pH, EC and Eh

Twenty grams of mine tailing were transferred in a baker, and 20 mL of dist. H_2_O were added. The mixture was homogenized and left to settle for 10 min or overnight. The pH was measured by a combined glass electrode and Hanna HI5522-02 multimeter. The redox potential and electroconductivity of suspension were measured using a combined platinum electrode with Ag/AgCl ref. (HI3230B, Hanna Instruments, Woonsocket, RI, USA) and conductometric cell (HI-763100, Hanna Instruments), respectively. The measured values of redox potential were adjusted to corresponding values vs. SHE by adding the standard potential of Ag/AgCl reference electrode (+224 mV S.H.E.). The particle size distribution was determined by sieving and determining the amount of material retained on a series of sieves with different sized apertures.

#### 2.1.2. Alkaline Reactivity

An accurately weighed sample of one gram was transferred into a plastic beaker. Twenty mL of 3.0; 6.5 or 10 M NaOH were added to each sample. The samples were agitated on reciprocal shaker at 100 min^−1^ for 10 min; 30 min; 60 min; 4 h; 24 h; 48 h; and 72 h. After the specified time, the samples were centrifuged for 5 min at 6000 rpm. A volume of 15.0 mL of the obtained supernatant was filtered and transferred into a 50.00 mL volumetric flask containing 10 mL of distilled water and 15 mL of conc. HNO_3_. The obtained solutions were diluted to volume by distill. H_2_O and sent to ICP-OES measurement of concentration of dissolved Al, Si and Ca. The concentrations of the solutions measured by ICP-OES were used to calculate the content of alkali dissolved Al, Si and Ca in mg/kg solid sample, taking into account the dilution factor.

#### 2.1.3. Total Heavy Metals Content of Copper Mine Tailing

The total heavy metal content was determined by ICP-OES after open acid digestion. A sample of 0.2–0.3 g was digested in aqua regia (HNO_3_ and HCl 1:3 *v*/*v*) (15 mL) and boiled for 25 min in a beaker covered with a watch glass. A total volume of 15 mL of the acid mixture was added in portions of 5 mL. The extract was filtered and diluted up to 50.00 mL with distilled water. The resulted solution was analyzed by ICP-OES.

#### 2.1.4. Water Leaching Characteristics

The leaching characteristics of the studied copper mine tailing were determined by a protocol based on EN 12457-2:2002 [23]. Ten grams air dried sample was mixed with 100 mL dist. water in a plastic beaker. The samples were agitated on a reciprocal shaker for 24 h at room temperature, 25 ± 2 °C. The dissolution process was followed by measuring pH and EC. In 24 h, the approximately stable results for pH and EC of supernatant were obtained; hence, the dissolution process was accepted to be completed. The samples were filtered to separate supernatant from solid residue. The obtained solutions were sent to ICP-OES for determination of elements and for UV-Vis for determination of nitrate, phosphate and sulfate ions. The obtained data for the concentration of dissolved ions in mg/L were calculated on a solid sample base and presented as mg/kg dry solid sample. Thus, the part of elements/ions of the sample soluble in water at pH of the sample for 24 h were estimated.

#### 2.1.5. Sequential Extraction of Heavy Metals

A modified four-step sequential extraction protocol based on the method proposed by the European Community Bureau Reference (BCR)—now Standard, Measurements and Testing Programme and modified by Gitari et al. [24]—was applied in the present study.

- Extraction step 1—determination of water/acid soluble and exchangeable fraction: 40 mL of 0.11 M HOAc was added to one g of accurately weighed dry sample (±0.0001 g). The mixture was shaken for 16 h on a reciprocal shaker (30 rpm) at 25 °C. The supernatant was filtered and collected in a dry vessel.
- Extraction step 2—determination of reducible or bound to Fe oxides fraction 2: 40 mL of 0.5 M NH_2_OH.HCl (adjusted to pH = 1.5 with HNO_3_) was added to the residue from step 1 and shaken for 16 h at 25 °C. The supernatant was filtered and collected in a dry vessel.
- Extraction step 3—determination of oxidizable or bound to sulphides and organic phases fraction: 10 mL of 8.8 M H_2_O_2_ was added to the residue from step 2 and digested for 1 h at 25 °C, and then for 1 h at 85 °C in a water bath with a second volume of H_2_O_2_. The solution was evaporated to a few milliliters. A total of 50 mL of 1 M NH_4_OAc (adjusted to pH = 2.0 with HNO_3_) was added to the residue. The suspension was shaken for 16 h at 25 °C. The supernatant was filtered and collected in a dry vessel.
- Extraction step 4—determination of residual or bound to silicates fraction: The final residue obtained from step 3 was dried at 120 °C for two hours. A total of 0.5 g of the dry residue was subjected to open vessel aqua regia digestion for 30 min without boiling. The solution was filtered and diluted to the final volume of 50 mL in a volumetric flask.

The obtained solutions were analyzed by ICP-OES for determination of heavy metals content.

#### 2.1.6. ICP-OES Analysis of Leachates

ICP-OES method for determination of heavy metals and metalloids in leachates from mine tailing was previously validated [25]. An ICP-OES spectrometer (Prodigy High Dispersion ICP-OES, Teledyne Leeman Labs, Hudson, NH, USA) with a dual-view torch, cyclonic spray chamber, and concentric nebulizer was used. The wavelengths free from spectral interferences at the studied concentration range were chosen [26]. Emission intensity of the analytes was a mean of at least three replicates. The analytical function was calibrated in two concentration ranges (5–100 μg/L or 1–10 mg/L). The calibration solutions were prepared by appropriate dilution of appropriate reference materials. Calibration curves and backgrounds correction were obtained by ICP-OES building software Salsa, Prodigy, HD ICP OES 2009, USA.

### 2.2. Geopolymers Obtaining and Characterization

Fly ash and mine tailing blended systems have been designed to analyze the influence of the Bulgarian copper mine tailings on the mechanical properties of geopolymers.

#### 2.2.1. Geopolymers Design and Preparation

The chemical composition of both raw materials has been analyzed by X-ray fluorescence (XRF) using an XRF S8 Tiger (Bruker GmbH, Karlsruhe, Germany) in order to establish a suitable activation method. The mineralogical composition of raw materials was determined by XRD analysis using X-ray diffractometer Empyrean, Panalytical with CuK radiation and building software. The step interval, integration time and angle interval used were 0.0530°; 53.8 s; and 5–80 2θ, respectively. Also, to assure experiment repeatability, the collected wastes have been dried until a constant weight (as described in [27]) was attained. As can be seen, both types of raw materials have Si and Al oxides in significant amounts. Also, the XRF analysis reveals a high content of Fe in the FA, which could negatively influence the mechanical performance of the geopolymers [28]. However, according to previous studies [29], it seems that in the case of fly ash-based geopolymers, the iron content has no influence. The FA also contains CaO, which could contribute to early strength development, along with decreasing setting time and curing temperature [30].

As activators, commercially available sodium silicate solution (S.C. KYNITA S.R.L., Valcea, Romania) and sodium hydroxide flakes (98% purity) from the same supplier were chosen. Prior to mixing with the Na_2_SiO_3_ solution, the NaOH flakes were dissolving in tap water at the desired concentration. The sodium silicate solution had a density of 1.52 g/cm^3^ and a pH of 11.5. Also, according to its quality certificate, the solution contains sodium silicate min. 44.8%, min. 31.10% SiO_2_, min. 13.70% Na_2_O and additives. Considering the chemical composition of the sodium silicate, the SiO_2_:Na_2_O ratio was determined as 2.27; therefore, 5.5 g of NaOH is necessary for each 100 g of sodium silicate in order to obtain the best alkaline activator [31]. Further, depending on the desired Na:Al ratio, the solid component is calculated based on the Na_2_O from the obtained activator and the Al_2_O_3_ from the aluminosilicate precursor [31].

To establish the influence of the MT addition on the mechanical properties of geopolymers, (i) three mixtures comprising different percentages of fly ash and mine tailings designated as B1 = 100% FA, B2 = 75% FA + 25% MT, and B3 = 50% FA + 50 MT (Table 2), (ii) three different liquid-to-solid ratios (0.70, 0.75, and 0.80), and (iii) three different Na:Al ratios (0.5, 0.75, and 0.1) were used. The Na:Al ratio was calculated considering the value of Al from the XRF analysis and the value of Na from the composition of the activator. The design of experiments with three factors and three levels is shown in Table 3. Considering the L9 orthogonal matrix Taguchi method, nine different mixtures were required to establish the influence of all factors involved. The mixture design is presented in Table 4.

**Figure 1:**
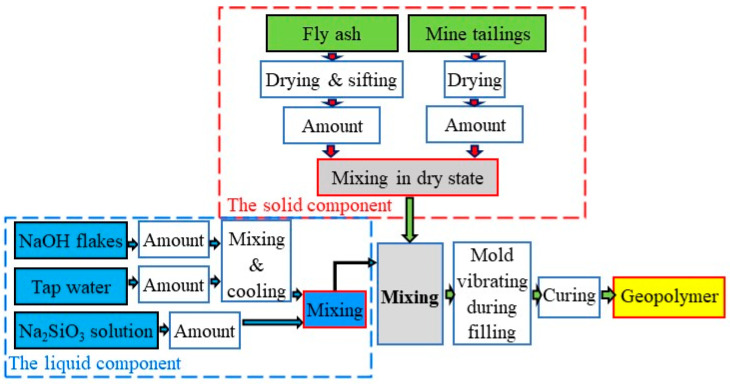
Schematic representation of the technological flow of geopolymers obtaining.

The procedure for obtaining the geopolymers consisted of the following steps: The raw materials (FA and MT) were dried, weighted, and mixed for 3 min (until a homogeneous mixture was obtained). Before mixing, the FA was dried, and only the particles lower than 100 µm in diameter were kept. During the process, sodium silicate and sodium hydroxide solution were mixed according to the composition. Afterwards, the liquid component (activator) was added over the solid component (raw materials) and mixed for 3 min to obtain a homogeneous mixture. The mixing of the components was conducted using a planetary mixer with variable speed, according to the EN 196-1:2016 standard [32]. A schematic representation of geopolymers obtained is presented in Figure 1. After mixing the liquid and the solid component, the obtained binder was poured into molds with sample dimensions of 40 mm × 40 mm × 160 mm, and their vibration was applied in order to obtain a uniform mixture with reduced air bubble content. The filled molds were then cured at room temperature (22 ± 2 °C) up to the testing age. For the first 24 h, the samples were covered with a plastic sheet in order to reduce the evaporation rate of the liquid, then kept in open air.

The most energy-efficient raw materials and processing procedures were examined to ensure that the developed technology is eco-friendly and has a low CO_2_ footprint. Moreover, in industrial use, both the drying and sifting stages can be removed since the water content can be evaluated through a moisture content, and then the amount already available in the raw materials can be reduced from the activator, while some impurities of large dimensions can exist in the raw materials will not significantly influence the properties of large, real-size, products.

#### 2.2.2. Geopolymers Analysis Methods

Both the compressive and flexural strengths of the developed materials have been tested at 14 and 28 days, respectively. The sample size and testing conditions have been followed according to SR EN 196-1:2016 requirements [32]. Accordingly, the flexural strength tests have been conducted on six specimens for each mixture (three have been tested at 14 days and three at 28 days) measuring 40 mm × 40 mm × 160 mm. The samples that resulted from flexural strength tests (two specimens split from the 40 mm × 40 mm × 160 mm specimen) have been used further to evaluate the compressive strength. As per standard requirements, a loading rate of 50 ± 10 N/s was chosen.

The microstructural analysis of the obtained geopolymers was conducted on the fracture surface after the mechanical tests using a scanning electron microscope (SEM) type FEI Quanta FEG 450 (FEI Company, Washington, DC, USA).

## 3. Results

### 3.1. Mine Tailing Caracterization

#### 3.1.1. Particle Size Distribution

**Figure 2:**
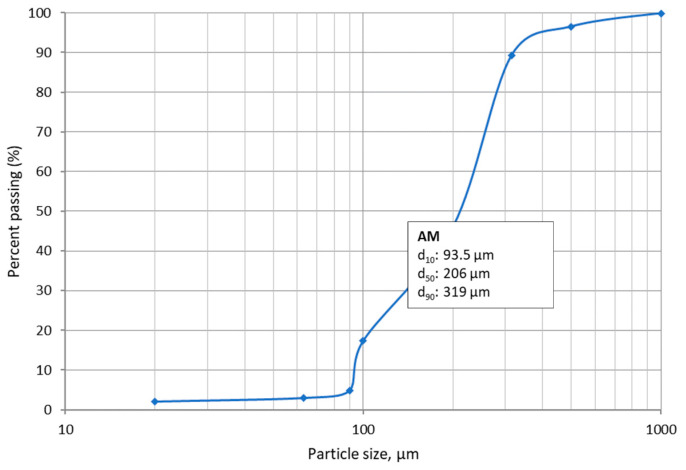
Particle size distribution of copper mine tailing.

The particle size distribution of copper mine tailing was studied as received without additional grinding. The particles were finer than 1250 µm. The main part of the particles (89%) was below 315 µm; almost half of the total amount of particles (46%) was below 200 µm. The fraction of particles with a size favorable for geoplymerization (<100 µm) was 17%. The finest fraction (between 90 and 63 µm) was 3%. The average values of particle size were 320, 210 and 90 µm for d_90_, d_50_ and d_10_, respectively (Figure 2). The particle size distribution curve was very steep, indicating a uniformly graded material [33]. Generally, the particle size distribution of the studied copper-mine tailing (90% below 0.3 µm) showed a fine sized material suitable for the geopolymerization process, according to the criteria proposed by Aseniero et al. [34]. However, the finer particle size was proven to increase the reactivity and to improves the geopolymers characteristics [35]. In this study, a relatively low percentage of the particles of mine tailing (17%) was below 100 µm, and the dimension was characterized with higher reactivity; thus, additional activation or blending with reach of aluminosilicates raw materials with fine particles was needed to obtain geopolymers with desired characteristics.

#### 3.1.2. Physicochemical Characteristics: pH, EC and Eh

**Figure 3:**
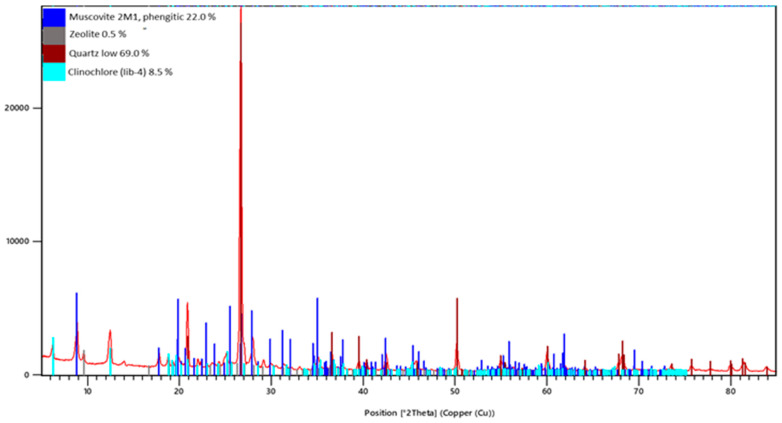
X-ray powder diffractogram of copper tailings sample. The main crystalline phases are presented in the Table 5.

The pH of the copper tailing at 1:1 solid-to-liquid ratio and 10 min settling time was 9.01, the electrical conductivity was 1796 µS/cm, and the redox potential was 122.4 mV (vs. Ag/AgCl). The alkaline pH was mainly due to the abundance of aluminosilicate minerals, such as clinochlore and muscovite found by XRD study (Figure 3). The redox potential of the aqueous supernatant was E = 366 mV (vs. SHE), indicating oxidative conditions in the slurry [36].

#### 3.1.3. Chemical and Mineralogical Composition

The chemical composition of copper mine tailing obtained by XRF is presented in Table 1. The results showed that the copper mine tailing was reached in SiO_2_ (66%) and Al_2_O_3_ (19%). The high content of Si and the significant content of Al indicated the presence of aluminosilicate minerals. The total content of alumina and silica was above 70%, which favored the geopolymerization process [37]. The ratio between Si/Al was calculated to be equal to 3. Moreover, the studied copper mine tailing contained a relatively low quantity of SO_3_ (3%), which was found to enhance the stability of the obtained geopolymer products [38,39,40]. Besides the Si and Al bearing minerals, elements such as Fe, Mg and Ca, can react and participate in the geopolymer matrix by forming other solid phases, which can affect the final properties of the structure [41]. In the studied sample, Fe_2_O_3_, MgO and CaO were between 2 and 3%. A relatively low content of Ca-bearing minerals (3% CaO) will affect the workability of the geopolymer paste, and additional sources of Ca should be added to the mixture. Between 1 and 4% of MgO, CaO, and K_2_O was found in the tailing. The presence of easily soluble basic components corresponded to high pH values measured in the water/tailing mixture (see Section 3.1.2). Although the sample contained Fe and S (around 3% as corresponding oxides), the high pH of the water leachate indicated that Fe was not in the form of Fe sulphide minerals [24].

The hazardous elements (Pb, Cu, Mn, Fe, As, Zn) were found at the levels of impurities (<1%) or traces (<0.1%). The concentrations of Pb, Cu and Mn were typical for industrial terrains.

An important prerequisite for raw materials is a higher content of Al_2_O_3_ and SiO_2_, preferably in amorphous reactive form [12,37,42]. The mineralogical composition of the studied copper mine tailing is presented on Figure 3 and Table 5. The primary minerals are quartz (SiO_2_) 69.0%, clinochlore ((Mg, Fe^2+^)_5_Al_2_Si_3_O_10_(OH)_8_) 8.5%, zeolite (SiO_2_) 0.5% and muscovite (KAl_2_(AlSi_3_O_10_) 22.0%. Alumosilicate minerals presented in the sample and the zeolite phase could be expected to favor the geopolymerization process, resulting in geopolymers with a high compressive strength [12,42,43].

#### 3.1.4. Alkaline Reactivity

**Figure 4:**
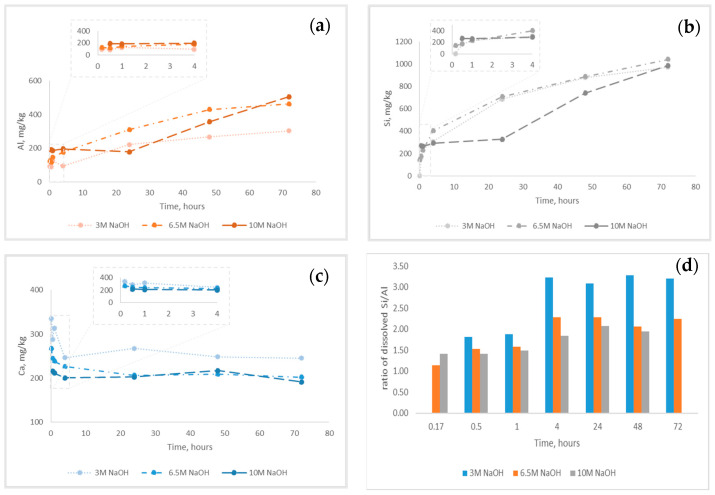
Alkali-dissolved Ca, Al and Si of the copper mine tailing as a function of time at different concentrations of NaOH: (**a**) Ca; (**b**) Al; (**c**) Si and (**d**) ratio of alkali-dissolved Si/Al as a function of alkali concentration and time of leaching. The alkaline reactivity is presented as mg dissolved component per kg solid sample. In-sets present the time intervals up to 4 h.

The alkaline reactivity of mine tailing was studied at different concentrations of alkali (3.0; 6.5 and 10 M NaOH). The level of dissolution of Ca, Al and Si in alkaline media was followed at different time intervals, starting from 10 min up to 72 h by the ICP-OES determination of the concentration of the studied components in the obtained solutions. The alkaline reactivity of studied mine tailing was estimated as mg dissolved component per kg solid sample. The results are presented in Figure 4a–c. The concentration ratio of dissolved Al to Si as a function of molarity of NaOH and time of leaching is presented on the Figure 4d.

Alkali dissolution of Al from mine tailing followed the pattern presented on Figure 4a. The concentration of dissolved Al constantly increased with time at each of studied concentration of NaOH. Increasing the alkali concentration up to 10 M solution in the first 48 h resulted in a decrease in the level of dissolved Al. At 72 h of contact between the solid and the liquid, the following results were obtained: 340 mg/kg in 3 M NaOH, 460 mg/kg in 6.5 M NaOH and 540 mg/kg in 10 M NaOH. The detailed results are presented in Table 3. At shorter periods (10 min to 4 h), the influence of alkali concentration was less pronounced: 93 mg/kg in 3 M; 125 mg/kg in 6.5 M and 190 mg/kg in 10 M NaOH.

Alkali dissolution of Si from the mine tailing followed the same trend as Al (Figure 4b). The concentration of dissolved Si constantly increased with time at each of the studied concentrations of NaOH. Up to 4 h of contact, the increased concentration of NaOH exerted a slight effect on the Si dissolution (Figure 4b inset): 306 mg/kg in 3 M NaOH; 403 mg/kg in 6.5 M NaOH and 330 mg/kg in 10 M NaOH. At the reaction times from 10 to 60 min, the quantity of dissolved Si slightly increased at each of the studied concentrations of alkali. The positive influence of the concentration of NaOH could be seen at these reaction times: highest dissolved Si was observed in 10 M NaOH. As can be seen from Figure 4b (inset), in the first 60 min, increasing the concentration of alkali resulted in a higher slope in the curve, which correspond to the increased rate of Si dissolution during the first contact. Moreover, in 10 M NaOH, the quantity of dissolved Si was approximately constant until 60 min: 270 mg/kg, indicating that the most reactive Si was dissolved in less than 10 min. More noticeable increases in the rate of Si dissolution in each of the studied alkali concentrations were observed at prolonged reaction times > 1 h.

As can be seen from Figure 4c, the concentration of dissolved Ca was higher in 3 M NaOH and decreased with increasing alkali concentration. The highest concentration of dissolved Ca was observed at the beginning of the contact between alkali and mine tailing, reaching a steady value after 4 h. At 10 min, the concentration of Ca varied from 330 mg/kg in 3 M NaOH to 210 mg/kg in 10 M NaOH. After 4 h until the end of the studied period, the concentration of dissolved Ca reached a steady value depending on the alkali concentration: 260 mg/kg in 3 M to 200 mg/kg in 6.5 and 10 M. After 4 h of contact between mine tailing and 6.5 and 10 M NaOH solution, the concentration of alkali did not influence the concentration of dissolved Ca. In contrast, 3 M NaOH still dissolve a higher amount of Ca.

Figure 4d presents the influence of alkali concentration and reaction time on the dissolved Si/Al ratio. As can be seen, increasing the alkali concentration resulted in a lower Si/Al ratio. This could be explained by the higher rate of Al dissolution in more concentrated NaOH solutions compared to Si. Hence, the alkali concentration exerted more pronounced effects on the dissolution of Al than on the dissolution of Si. The trend was observed in the whole studied period from 10 min to 72 h. It should be noted that the highest difference in Si/Al ratio was observed when increasing concentrations from 3 to 6.5 M. Higher concentrations of alkali showed slightly leveling effect.

The results presented in the Table 6 demonstrated the alkaline solubility of the main geopolymer forming components in the studied copper mine tailing. The concentration of dissolved Al in mg per kg dry solid sample was taken as a base for ratio calculations. The concentration ratio of dissolved Ca and Si followed the same pattern: the increased concentration of NaOH resulted in lower Ca to Al and Si to Al ratio. The same trend in Ca to Al ratio is observed regarding the contact time. The findings could be explained by the increased concentration of Al with increasing the concentration of alkali and the contact time. In contrast, the Si to Al ratio increased with shorter reaction times, reaching state value after 4 h. A possible explanation could be found in the faster rate of dissolution of Si than of Al in the studied sample in the beginning of contact between alkali and solid material; after 4 h, the rate of dissolution of both components is almost equal. At shorter reaction times (<60 min), the Al/Si ratio is not influenced by the concentration of NaOH and is found to be approximatively 1:2 in all studied concentrations. More pronounced effects of alkali were observed at longer contact times (>60 min). In 3 M NaOH, the Al/Si ration was 1:3 and lowered to 1:2 in 10 M alkali.

#### 3.1.5. Heavy Metals Content and Mobility in Aqueous Medium

Table 7 presents the chemical composition of copper mine tailings determined by ICP-OES after aqua regia digestion and the composition of the generated aqueous leachate. The obtained results allowed for the environmental impact of mine tailing to be estimated and give some indication about the chemical composition of generated plume after interaction between the tailing and rainwater [24]. The generated leachates from the copper mine tailings were alkaline with pH 9.53 and were stable in the studied period of 24 h. The electrical conductivity of leachate was 750 µS/cm after 2 h and reached 850 µS/cm after 24 h.

The concentrations of Cd and Co in the generated leachate were below <0.005 mg/L, and Cr and As were below 0.010 mg/L. The highest concentrations in leachate were observed for (in decreased order) Fe > Ca > Mg > Al. Additionally, the concentrations were calculated in a mg/kg air dried sample to present the concentrations of ions in solid-sample dissoluble in water at sample pH and accordingly were capable of moving into the aqueous solution. The results showed that less than 0.5 mg/kg of Cd, Co, Cr and As in copper mine tailing were in water soluble form; the main part of these components should be in non-labile geochemical fractions. Fe, Al, Ca and Mg showed the highest water-soluble concentrations: 2462, 694, 389 and 699 mg/kg, respectively, and govern the electrical conductivity of the leachate. The concentration in leachate of the studied anions decreased in the following order SO_4_^2−^ > NO_3_^−^ > PO_4_^3−^.

#### 3.1.6. Heavy Metals Distribution in Mine Tailing Fractions

Heavy metal distribution in geochemical fractions of copper mine tailing was studied by a BCR sequential extraction procedure, including the additional step of the analysis of the residual fraction. The heavy metal fractionalization was strongly correlated with their availability and possible toxicity to the environment. The sum of the heavy metals in exchangeable fraction (F1), iron and manganese oxide fraction (F2) and organic matter and reducible fraction (F3) presented labile species, which were susceptible to dissolution and transferred into liquid phase. The residual fraction (F4) presented the non-labile phase. The following metals were studied: Fe, Cu, Zn, Mn, Cd, Ni, Pb and As. The results showed that Cd, Ni and As were below method LOQ (<0.5 mg/kg) in the studied copper tailing sample. The total content of heavy metals showed that Fe is the most abandoned element, followed by (in decreasing order) Cu, Mn, Zn and Pb. The sum of F1, F2 and F3 was considered as a mobile part of the heavy metals. The following decreasing trend in concentrations was observed: Fe > Cu > Mn > Pb > Zn. Considering the first three fractions, it could be seen that all of the studied metals showed the highest concentration in liquid phase in the F3: organic matter and reducible fraction. The residual fraction was considered to contain the non-labile forms of the heavy metals. In this fraction the elements were arranged as follows: Fe > Cu > Mn > Zn > Pb. The results showed that the highest content of the studied hazardous elements was found in the residual fraction, indicating the limited mobility and bioavailablity of hazardous components of the copper mine tailing.

The partitional pattern of Fe showed that the main part of Fe in the copper tailing (approximatively 50%) was in a mobile form (sum F1 + F2 + F3). The highest concentration was observed in Fraction 3: organic matter and reducible fraction.

The concentration of dissolved Cu in sequential fractions decreased in the following order: F3 > F1 > F2. A total of 77% of the total Cu was found in the mobile fractions, and 23% was found in the residual fraction. The main part of the Cu species was extracted in the F3 organic matter and reducible fraction. The minor part of Cu was bound to Fe- and Mn oxides.

The main part of Zn was found in the residual fraction, indicating its low lability and corresponding bioavailability. Around 36% of the total Zn in the copper mine tailing was extracted in the sequential extraction steps. The highest quantity of Zn was bound to organic matter and the reducible fraction. The quantity of Zn in exchangeable fraction and Zn bound to Fe and Mn oxide were approximatively equal.

The main part (60%) of Mn in the copper mine tailing was found in the non-libile form in the residual fraction. The highest concentration of dissolved Mn was observed in the F3 fraction, and the lowest was in the F2 fraction. Around 12% of the total Mn was observed in the F1 exchangeable fraction, 6% in F2 and 23% in F3. The results indicated the low mobility of Mn in the studied sample.

The concentration of dissolved Pb followed the pattern F4 > F3 > F2 > F1. The main part (60%) of Pb in the sample was bound into the mobile fractions. However, it could be observed that less than 10% of the available Pb was found in the easily exchangeable fraction.

### 3.2. Geopolymers Analysis

#### 3.2.1. Mechanical Properties

**Figure 5:**
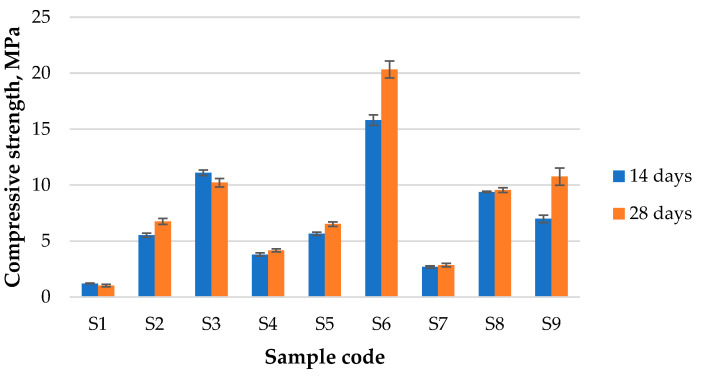
The compressive strength of the obtained geopolymers.

**Compressive strength.** Figure 5 shows the evolution of compressive strength from 14 to 28 days for the obtained geopolymers. As can be seen, the mixture specific to S1 did not perform too well at this test, being one of the samples with almost the same lost value at 28 days. In this case, S6 exhibited the optimum composition despite the aging time, while S1 and S3 showed a slight decrease after 28 days of curing. The increase in compressive strength was in the range of 22 to 54%; the highest evolution was presented by S9, while the lowest was shown by S8. Overall, it can be observed that the compressive strength depends on the raw material mixture and the Na:Al ratio; higher ratios result in better compressive strength. A replacement of FA with MT seems to promote the best compressive strength, despite the Na:Al ratio. A 25 wt.% replacement of FA with MT showed the optimum replacement, yet at 50 wt.% of MT, the compressive strength of the blended geopolymer is superior to that based on FA at almost all testing ages and Na:Al ratios. Moreover, all mixtures showed a low deviation value, which confirms that the obtained samples had a homogenous structure and composition.

**Figure 6:**
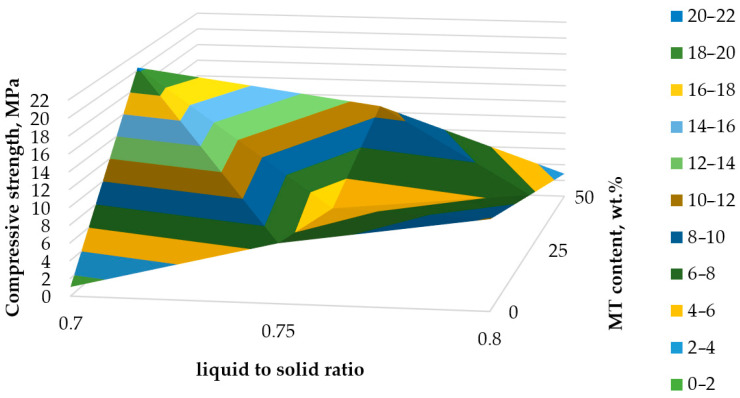
Influence of liquid-to-solid ratio and MT content on compressive strength at 28 days.

To better understand the influence of the obtained parameters on the mechanical properties of the obtained geopolymers, 3D plots were drawn. Figure 6 shows the influence of MT content and the liquid-to-solid ratio on the compressive strength. As can be seen, higher liquid content will result in poor mechanical properties. The optimum mixture is the one with 25 wt.% MT and a 0.7 liquid-to-solid ratio. This behavior could be related to activator loss during curing. The OH^−^ from the activator, especially from the NaOH solution, is responsible for the leaching process of Si^4+^ and Al^3+^ ions; therefore, a loss of activator will also result in a loss of the necessary ions to assure the dissolution of the aluminosilicate material [45]. Moreover, during this drying stage, the water evacuation will also promote crack formation [46]. The samples with only FA as a precursor showed an increase in compressive strength with the increase in liquid-to-solid ratio, probably due to the nature of the FA particles sponge-like structure, which will absorb the activator and release it much slower compared to the compact particles from MT [47].

**Figure 7:**
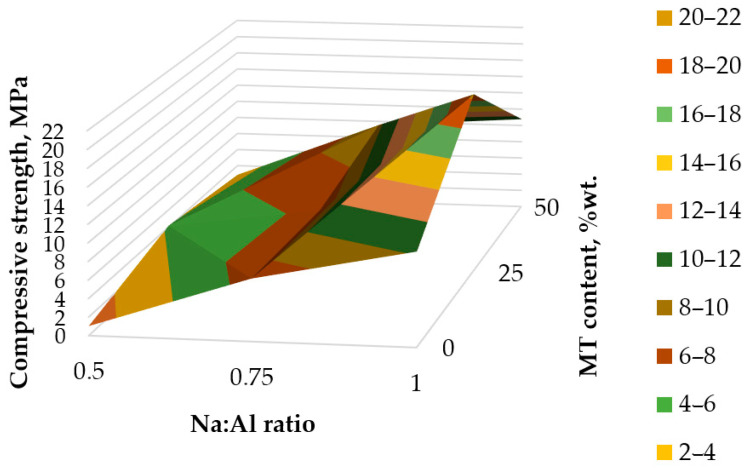
Influence of Na:Al ratio and MT content on compressive strength at 28 days.

Considering the dependence between the amount of MT and the Na:Al ratio (Figure 7), it can be observed that the 25 wt.% replacement of FA with MT will not produce the best compressive strength, despite the activating conditions. For example, at a Na:Al ratio of 0.75, the mixture with 50 wt.% MT performed better than all other geopolymers. Even though the increase in Na:Al ratio resulted in better mechanical properties for all mixtures, only the FA-based geopolymer seems to present a continuous trend from a Na:Al of 0.5 to a Na:Al of 1. In the case of the other mixtures, the Na:Al increase seems to have a negative effect at 0.75 for the geopolymer with 25 wt.% MT or at 1 for the geopolymer with 50 wt.% MT.

The data used to create the 3D graphs from Figure 6 and Figure 7 are presented in Supplementary File S1.

**Figure 8:**
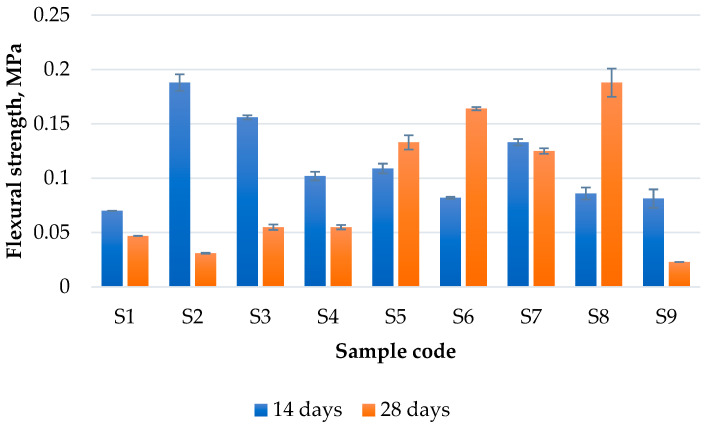
The flexural strength of the obtained geopolymers.

**Flexural strength.** The flexural strength of the obtained geopolymers decreased in most of the cases from 14 days to 28 days (Figure 8), except for samples S5, S6 and S8, where a significant improvement in flexural strength can be observed. For example, from 14 to 28 days, the value of S8 and S6 increased almost two times, and the lowest increase was that of S5, which is approximately 20% higher. As observed, all mixture with FA as raw materials showed a decrease in flexural strength, probably due to the high Ca content that will react faster than Al, while at latter stages, the Si will start reacting, resulting in a much more brittle structure [48]. On the contrary, most of the samples that have MT content showed an increase in flexural strength over time, probably due to a lower concentration of Ca from the system. Considering the Na:Al ratio, it can be seen that only the mixtures with a Na:Al of 0.5 showed a decrease in flexural strength over time. At 14 days, the optimum composition was that of S2, while at 28 days, S8 showed the highest value.

#### 3.2.2. Microstructural Analysis of the Obtained Geopolymers

**Figure 9:**
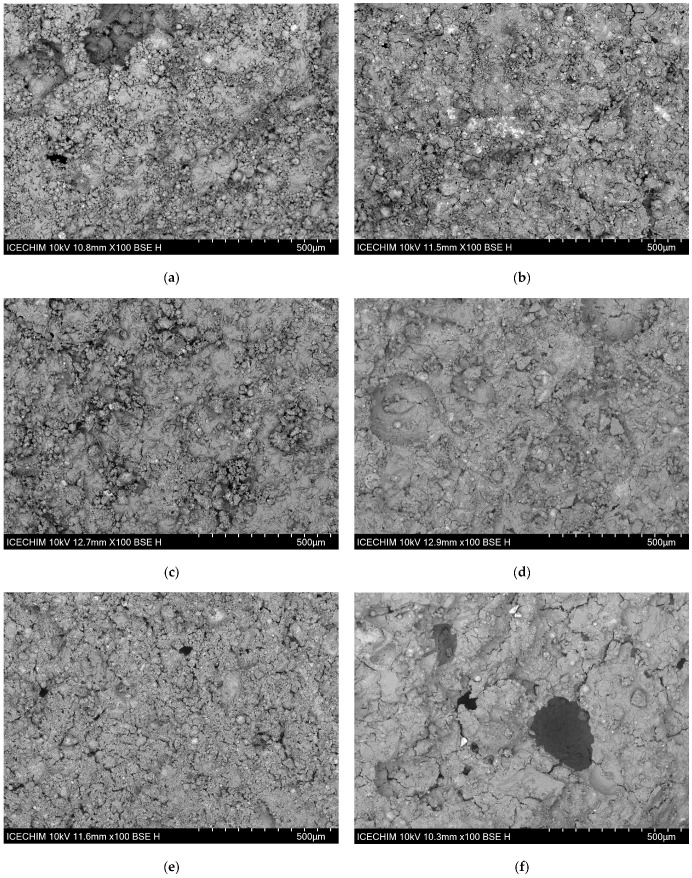
Microstructural analysis of the obtained geopolymers: (**a**) sample S1; (**b**) sample S3; (**c**) Sample S4; (**d**) sample S6; (**e**) sample S7; (**f**) sample S9.

To emphasize the link between the morphological characteristics of the created materials and their mechanical performances, the combination with the lowest compressive strength was compared to the mixture with the highest value for each mixture of raw materials. As shown in Figure 9, the microstructure of the S1 geopolymer (Figure 9a) contains multiple unreacted coal ash particles, particularly those with large diameters. Moreover, there are large areas that were not dissected, which act as defects, resulting in a poor structure in terms of mechanical properties. Compared to the S1 sample, the sample with a Na:Al ratio of 1 and FA as raw material, i.e., S3, showed a much more compact matrix with better dissolution; however, the matrix also exhibits multiple pores and cracks distributed all over the analyzed surface (Figure 9b). When mine tailings are introduced in the mixture, the morphology of the structure significantly changes; the matrix shows very few cracks, but there are still some areas where unreacted FA particles can be observed (Figure 9c). The samples with the highest compressive strength, i.e., S6, show a substantially more compact matrix than all other mixtures (Figure 9d). Some unreacted FA particles can be observed, but most of them are embedded in the compact matrix. By increasing the MT content to 50 wt.%, the dissolution of the raw materials seems to decrease (Figure 9e), while a significant increase in the number of cracks occurs (Figure 9f).

**Figure 10:**
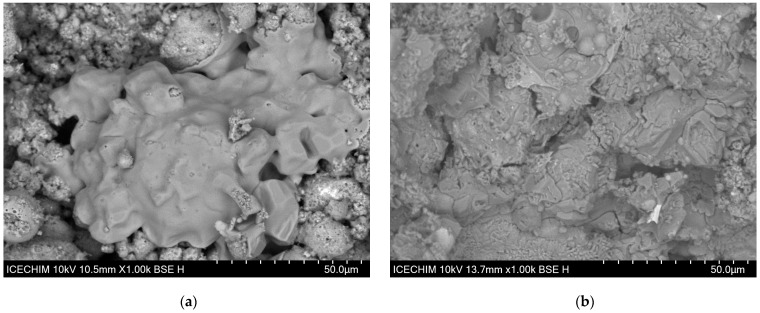
The microstructure of the obtained geopolymers at 1k× magnification: (**a**) sample S1; (**b**) sample S6.

As can be seen in Figure 10, at higher resolution, the differences between the best (sample S6) and the worst (sample S1) samples in terms of compressive strength are even more visible. Compared to S6 (Figure 10a), the microstructure of S1 (Figure 10b) shows activation only in limited areas (the smooth area from the image), while high areas show morphology specific to the nonreacted particles. However, in the case of S6, most particles seem to be activated, while those that remain unreacted are embedded in the matrix.

Considering the composition of the compared mixture, it can be stated that higher Na:Al ratios will increase the dissolution of the aluminosilicate source and increase the compactness of the matrix, promoting better mechanical properties.

## 4. Discussion

A detailed characterization of the Bulgarian copper mine tailing from Assarel-Medet as a precursor for geopolymer obtaining was reported for the first time. The chemical and mineralogical composition, leaching behavior, alkaline reactivity amd heavy metal distribution in the geochemical phases of mine tailing were studied. The conclusions drawn were focused on its utilization as a raw material for obtaining geopolymers.

The chemical composition of the studied copper mine tailings showed their perspective to be reused as raw materials for obtaining geopolymers (Table 1). The high content in Si and significant content in Al-bearing minerals are favorable for the geopolymerization process [37]. The ratio of Si/Al in the copper mine tailing was in the optimal ratio of 1–3 [12,24,35,49,50] thus making the studied tailing a promising precursor for geopolymers. However, an additional source of Al could be added to enhance the geopolymerization process and improve the characteristics of the obtained products [24,51]. In a recent review, Lazorenko et al. summarized the chemical composition of various mine tailings used for geopolymers. The SiO_2_ content in copper mine tailing from different sources varied between 28 and 65%, and for Al_2_O_3_ from 4 to 14% [52]. Comparing the composition of the studied material, it could be concluded that it is a suitable precursor for geopolymerization, having the benefit of higher Al content.

The study of mineralogical composition proved the presence of alumosilicate minerals (muscovite, clinochlore), as well as SiO_2_ in the zeolite and quartz phases. The presence of alumosilicates in the tailings could be expected to exert a neutralizing or pH buffering effect on generated leachates [24], which will mitigate the environmental effect during rainfall. The study of the alkaline reactivity of copper mine tailing (Figure 4) revealed that the alkali-leachable Si и Al were between 0.2 and 0.5% from the total content of SiO_2_ and Al_2_O_3_ in the raw material. Hence, the low extent of dissolution of crystalline phases in the studied conditions 3, 6.5 and 10 M NaOH at different times up to 72 h was observed. The results were in support of the findings of Cristello et al., who demonstrated that crystalline phases in copper tailing were inert [14]. The ratio of reactive Si and Al in copper tailing (Table 6) was also studied, as it was known to influence the formation of geopolymer networks [14,15,17]. However, a convincing answer for which ratio was optimal for designing a geopolymer product with the intended characteristics and application could not be found. The reason was that additional sources of reactive Si were added to the precursor mixture during the obtaining of geopolymer material: (i) sodium silicate and (ii) fly ash. Thus, the properties of the obtained materials depended on the complex combinations of parameters that govern the alkali activation process [5,15,53]. It was observed that increasing the molarity of NaOH in activator solution resulted in a higher strength of the obtained geopolymer materials. The results were in line with previous studies [12,14,15]. It could be explained only partially by the increased dissolution rate of aluminosilicates, and accordingly, the concentrations of reactive Si and Al, as fly ash addition, could positively influence the development of the geopolymer network [5,14].

Additionally, the studied mine tailing contained fine particles; hence, the mine tailing possessed a potential to be an appropriate raw material for geopolymerization.

The results from sequential extraction demonstrated the low potential environmental risk, as it was found that the main part of hazardous heavy metals was immobilized in the non-labile phase. It could be concluded that the copper mine tailing is a promising precursor for obtaining building products based on the geopolymerization process. Additionally, due to the encapsulation of heavy metals and other environmentally contaminants in the geopolymer matrix, the mobility of hazardous compounds could be expected to be lower in the final geopolymer product [40].

The chemical composition, the behavior in alkaline media, the leaching characteristics of the studied copper mine tailing, as well as the fractionalization of the potential environmental contaminants demonstrated its high potential for the valorization of the tailings in the form of geopolymer products for building engineering.

## 5. Conclusions

Copper mine tailings from the Assarel Concentrator Plant (Bulgaria) can be successfully used as precursors for geopolymers. The replacement of 25 wt.% of fly ash with mine tailings promoted better compressive strength. By increasing the replacement of FA with MT to 50 wt.%, a significant decrease in compressive strength can be observed, especially for the mixture with a Na to Al ratio of 1.

From the three mixtures of raw materials, three different liquid-to-solid ratios, and three different Na-to-Al ratios, the system with 75 wt.% FA, 25 wt.% MT, a liquid-to-solid ratio of 0.7, and a Na-to-Al ratio of 1 exhibits the highest compressive strength at 28 days of curing. From a flexural strength point of view, the system with 50 wt.% MT, a liquid-to-solid ratio of 0.7, and a Na-to-Al ratio of 0.75 was optimum, but at a very low difference from the system that also exhibited the highest compressive strength. Therefore, from all nine mixtures, it can be considered that the one that had the highest compressive strength is optimal from a mechanical properties point of view.

The microstructural analysis showed a clear relationship between the homogeneity of the matrix and the mechanical performance of the mixture. Accordingly, it was observed that compact matrixes with fewer cracks and unreacted particles would perform better.

## Figures and Tables

**Table 1:** The oxide chemical composition of the raw materials.

Oxide		SiO_2_	Fe_x_O_y_	Al_2_O_3_	CaO	SO_3_	K_2_O	TiO_2_	Na_2_O	BaO	SrO	ZrO_2_	MgO	P_2_O_5_	Oth.
FA	wt.%	41.20	21.80	15.90	12.30	3.50	2.70	1.20	0.67	0.34	0.18	0.06	-	-	<0.05
MT	wt.%	65.50	2.67	19.20	1.33	3.13	4.70	0.32	0.97	-	0.03	0.01	1.90	0.19	<0.05

**Table 2:** The mix design of solid components for geopolymers.

Mixture Code	Composition of Mixtures, wt.%	
	FA	MT
B1	100	0
B2	75	25
B3	50	50

**Table 3:** Experimental factors and levels.

Factor	Level 1	Level 2	Level 3
A. Binder content	B1	B2	B3
B. Na:Al ratio	0.5	0.75	1
C. Liquid-to-solid ratio	0.7	0.75	0.8

**Table 4:** Mixing combinations of geopolymer samples.

Sample Code	S1	S2	S3	S4	S5	S6	S7	S8	S9
Factor A	B1	B1	B1	B2	B2	B2	B3	B3	B3
Factor B	0.5	0.75	1	0.5	0.75	1	0.5	0.75	1
Factor C	0.7	0.75	0.8	0.75	0.8	0.7	0.8	0.7	0.75

**Table 5:** The main crystalline phases in the studied copper mine tailing by XRD.

Reference	Crystalline Phase	Composition
98-008-3849	Quartz low	SiO_2_
98-008-4262	Clinochlore (IIb-4)	Mg_5_Al(AlSi_3_O_10_) (OH)_8_
98-017-0492	Zeolite	SiO_2_
98-007-7495	Muscovite 2M1, phengitic	K(Al,Mg)_2_(OH)_2_(Si,Al)_4_O_10_

**Table 6:** Ratio of dissolved Ca/Al/Si in copper mine tailing as a function of alkali concentration and reaction time.

Concentration of NaOH, M	Reaction Time, h	Dissolved Ca/Al/Si Ratio ^1^
3	10′	3.6/1/0
	30′	3.2/1/1.8
	60′	2.5/1/2
	4 h	2.6/1/3.2
	24 h	1.2/1/3.1
	48 h	0.9/1/3.3
	72 h	0.8/1/3.2
6.5	10′	2.1/1/1.1
	30′	2.1/1/1.5
	60′	1.6/1/1.6
	4 h	1.3/1/2.3
	24 h	0.7/1/2.3
	48 h	0.5/1/2.1
	72 h	0.4/1/2.2
10	10′	1.1/1/1.4
	30′	1/1/1.5
	60′	1.1/1/1.8
	4 h	0.6/1/2.1
	24 h	0.4/1/1.9
	48 h	0.4/1/1.9
	72 h	1.1/1/1.4

^1^ Concentration of dissolved Al in mg/kg dry material was taken as 1 for calculation of the ratio.

**Table 7:** Chemical composition of copper mine tailing and generated aqueous leachate.

Component	Concentration in Mine Tailing, mg/kg	Concentration in Leachate, mg/L	MPL ^1^, mg/L	Component	Concentration in Mine Tailing, mg/kg	Concentration, mg/L	MPL ^1^, mg/L
Ca	9491	123	150	Cd	<0.5	<0.010	0.005
Mg	699	2.93	80	Ni	6.700	<0.010	0.02
Na	278	9.28	200	Co	<0.5	<0.005	
Al	694	0.27	0.2	Pb	traces	traces	0.010
Fe	18,629	<0.005	0.2	NO_3_^−^		1.0	0.05
Cu	375	<0.005	0.002	SO_4_^2−^		370	250
Zn	72.5	<0.010	4	PO_4_^3−^		<0.5	0.5
Mn	186	<0.010	0.50	Cl^−^			250
As	<2	<0.010	0.010	pH		9.53	
S	3734	198		EC, µS/cm		750	
Cr	<0.5	<0.010	0.05				

^1^ Maximum permissible level according to the Bulgarian regulation for the quality of drinking water [44].

## Data Availability

Data are contained within the article and supplementary materials.

## Supplementary Materials

The supporting information can be downloaded at: https://www.mdpi.com/article/10.3390/ma17030542/s1, Table S1 (Table for Figure 6): Influence of liquid-to-solid ratio and MT content on compressive strength; Table S2 (Table for Figure 7): Influence of Na:Al ratio and MT content on compressive strength at 28 days.
