# Supplementary material for: Characterization of Bulgarian Copper Mine Tailing as a Precursor for Obtaining Geopolymers
Source: Materials (Basel). 2024 Jan 23;17(3):542. doi: 10.3390/ma17030542 (PMC10856451; doi:10.3390/ma17030542)
Supplement: Supplementary file 1 [file materials-17-00542-s001.zip › materials-2815113-supplementary.pdf]

Supplementary File S1

Data tables used to create the 3D graphs

Table S1. Influence of liquid-to-solid ratio and MT content on compressive strength at 28 days

		liquid to solid ratio		
		**0.5**	**0.63**	**0.75**
**MT content, wt.%**	**25**	1.21	5.35	11.10
	**50**	3.80	5.66	15.82
	**75**	2.69	9.40	6.99

* The values in red are the mean values of compressive strength.

Table S2. Influence of Na:Al ratio and MT content on compressive strength at 28 days.

		Na:Al ratio		
		**0.5**	**0.63**	**0.75**
**MT content, wt.%**	**25**	1.21	5.54	11.10
	**50**	15.82	3.80	5.66
	**75**	9.40	6.99	2.69

* The values in red are the mean values of compressive strength.
